# Integrated analysis and exploration of potential shared gene signatures between carotid atherosclerosis and periodontitis

**DOI:** 10.1186/s12920-022-01373-y

**Published:** 2022-10-31

**Authors:** Youjie Zeng, Si Cao, Minghua Chen

**Affiliations:** Department of Anesthesiology, Third Xiangya Hospital, Central South University, 410013 Changsha, Hunan China

**Keywords:** Carotid atherosclerosis, Periodontitis, Bioinformatics, Hub genes, Gene expression omnibus, Differentially expressed genes, Biomarker, Crosstalk

## Abstract

**Background:**

Increasing evidence has suggested an association between carotid atherosclerosis (CAS) and periodontitis (PD); however, the mechanisms have not been fully understood. This study aims to investigate the shared genes and molecular mechanisms underlying the co-pathogenesis of CAS and PD.

**Methods:**

Gene Expression Omnibus (GEO) datasets GSE100927 and GSE10334 were downloaded, and differentially expressed genes (DEGs) shared by both datasets were identified. The functional enrichment analysis of these overlapping DEGs was then conducted. A protein-protein interaction (PPI) network was created using the STRING database and Cytoscape software, and PPI key genes were identified using the cytoHubba plugin. Then, weighted gene co-expression network analysis (WGCNA) was performed on GSE100927 and GSE10334, and the gene modules most correlated with CAS and PD were identified as key modules. The genes in key modules overlapping with PPI key genes were determined to be the key crosstalk genes. Subsequently, the key crosstalk genes were validated in three independent external datasets (GSE43292 [CAS microarray dataset], GSE16134 [PD microarray dataset], and GSE28829 [CAS microarray dataset]). In addition, the immune cell patterns of PD and CAS were evaluated by single-sample gene set enrichment analysis (ssGSEA), and the correlation of key crosstalk genes with each immune cell was calculated. Finally, we investigated the transcription factors (TFs) that regulate key crosstalk genes using NetworkAnalyst 3.0 platform.

**Results:**

355 overlapping DEGs of CAS and PD were identified. Functional enrichment analysis highlighted the vital role of immune and inflammatory pathways in CAS and PD. The PPI network was constructed, and eight PPI key genes were identified by cytoHubba, including CD4, FCGR2A, IL1B, ITGAM, ITGAX, LCK, PTPRC, and TNF. By WGCNA, the turquoise module was identified as the most correlated module with CAS, and the blue module was identified as the most correlated module with PD. Ultimately, ITGAM and LCK were identified as key crosstalk genes as they appeared both in key modules and PPI key genes. Expression levels of ITGAM and LCK were significantly elevated in the case groups of the test datasets (GSE100927 and GSE10334) and validation datasets (GSE43292, GSE16134, and GSE28829). In addition, the expression of multiple immune cells was significantly elevated in PD and CAS compared to controls, and the two key crosstalk genes were both significantly associated with CD4 T cells. Finally, SPI1 was identified as a potential key TF, which regulates the two key crosstalk genes.

**Conclusion:**

This study identified the key crosstalk genes and TF in PD and CAS, which provides new insights for further studies on the co-morbidity mechanisms of CAS and PD from an immune and inflammatory perspective.

**Supplementary Information:**

The online version contains supplementary material available at 10.1186/s12920-022-01373-y.

## Background

The leading pathological process of cardiovascular disease, atherosclerosis, has become a major cause of disability and mortality throughout the world [1]. The carotid artery is one of the primary early onsets of atherosclerosis in seemingly healthy individuals [2]. There is currently a substantial global burden of carotid atherosclerosis (CAS), with 21% of patients aged 30–79 years suffering from carotid plaque [3]. Furthermore, studies have shown that CAS contributes to new occurrences of ischemic stroke [4, 5].

Periodontitis (PD) is a chronic inflammatory disease that affects the supporting tissues around the teeth [6]. Increasing evidence suggests an association between PD and CAS [7, 8]. The risk factors for PD, such as smoking, diabetes, and obesity [9], are also associated with atherosclerosis [3]. In addition, PD serves as an independent risk factor for atherosclerotic cardiovascular disease [10]. The majority of studies indicate that systemic inflammation plays a significant role in PD and CAS [11]. Inflammatory mediators (e.g., C-Reactive Protein, matrix metalloproteinases, fibrinogen, and other hemostatic factors) are elevated by PD, thus accelerating the progression of CAS through oxidative stress and inflammatory dysfunction [12]. Further, bacteremia attributed to PD can promote direct targeting of bacteria to the distal carotid artery, thereby triggering CAS [13]. Carotid atheromatous plaques obtained during carotid endarterectomy were found to contain DNA of multiple periodontitis-associated bacteria, the most common of which was Porphyromonas gingivalis, followed by Aggregatibacter actinomycetemcomitans, Tannerella forsythia, Eikenella corrodens, Fusobacterium nucleatum, and Campylobacter rectus [14]. These findings suggest a strong linkage between CAS and PD, while molecular mechanisms and pathological interactions are not fully understood.

In this study, we analyzed the publicly available datasets of the GEO database. First, we identified the differentially expressed genes (DEGs) of CAS and PD in test datasets (GSE100927 [CAS] and GSE10334 [PD]) and obtained overlapping DEGs. Subsequently, we performed the functional enrichment analysis of overlapping DEGs. Next, we construct the PPI network using the STRING database. Then, we identified PPI key genes via the Cytoscape software. Furthermore, we performed weighted gene co-expression network analysis (WGCNA) on GSE100927 and GSE10334, thereby screening the most correlated gene modules with CAS and PD. The intersecting genes between PPI key genes and key modules were defined as key crosstalk genes. These key crosstalk genes were verified in the validation datasets GSE43292 (CAS microarray dataset), GSE16134 (PD microarray dataset), and GSE28829 (CAS microarray dataset). In addition, we evaluated the immune cell patterns of PD and CAS by single-sample gene set enrichment analysis (ssGSEA), and calculated the correlation of key crosstalk genes with each immune cell. Finally, we explored the transcription factors (TFs) regulating the key crosstalk genes and finally obtained TFs that were generally up-regulated in CAS and PD. Our study may shed new light on the shared pathogenesis of CAS and PD.

## Materials and methods

### Data source

Expression data of CAS and PD were obtained from the Gene Expression Omnibus (GEO) database (https://www.ncbi.nlm.nih.gov/geo/) [15]. The search strategy of this study: (1) the gene expression profiles were produced by array; (2) PD dataset samples were obtained from gingival tissue, and CAS dataset samples were obtained from carotid plaque; (3) datasets contain samples of control groups; (4) samples are sourced from Homo sapiens. The dataset GSE100927 is based on the GPL17077 Agilent-039494 SurePrint G3 Human GE v2 8 × 60 K Microarray 039381. It included 29 carotid atherosclerotic lesion samples and 12 normal carotid samples. GSE10334 was generated using GPL570 [HG-U133_Plus_2] Affymetrix Human Genome U133 Plus 2.0 Array, which contained 183 gingival tissue samples affected by PD and 64 unaffected control gingival tissue samples. Details of the validation dataset are described in the “Validation of Key Crosstalk Genes in Independent External Datasets” section. The data we analyzed were publicly available; therefore, no ethics committee approval or informed consent was required.

### Differentially expression analysis

Analyses based on R packages were carried out on R software (version 4.1.1; https://cran.r-project.org/). DEGs were identified by the “Limma” R package [16]. First, the data were normalized using the normalizeBetweenArrays function. Subsequently, the probe IDs were transformed into official gene symbols. Then, the log_2_ fold change and adjusted P-value of each gene were calculated by the lmFit and eBayes functions. The genes with adjusted P-value < 0.05 and |log_2_ fold change| > 0.5 were identified as DEGs. Finally, we defined overlapping DEGs as genes that were simultaneously up-regulated or simultaneously down-regulated in both GSE100927 and GSE10334 datasets. The “ggvenn” R package (https://cran.r-project.org/web/packages/ggvenn/) was performed to plot the Veen diagram for overlapping DEGs.

### Enrichment analyses of overlapping DEGs

To further understand the function of the overlapping DEGs in CAS and PD, we performed Gene Ontology biological process (GO_BP) enrichment analysis [17, 18], Kyoto Encyclopedia of Genes and Genomes (KEGG) pathway enrichment analysis [19–21], and Reactome pathway enrichment analysis [22] using the DAVID Bioinformatics Resources (https://david.ncifcrf.gov/) [23]. Terms with FDR < 0.05 were considered to be significantly enriched. The downloaded results were visualized through the Sangerbox data analysis platform (http://sangerbox.com/) [24].

### Protein-protein interaction (PPI) network construction

To identify interactions between overlapping DEGs, we constructed a PPI network of the overlapping DEGs using the STRING database (Version: 11.5) (http://string-db.org/). This database enables researchers to construct a functional association network of uploaded proteins of an organism based on three aspects: prior knowledge, computational interaction predictions and direct lab experiments [25]. We obtained the interaction network of overlapping DEGs with the “minimum required interaction score” parameter set to 0.4. Subsequently, we imported the network into Cytoscape software for visualization [26]. MCODE plugin app was used to filter clusters with high connectivity, thus dividing the PPI network into several clusters (default parameters: Degree Cutoff = 2; Node Score Cutoff = 0.2; K-Core = 2; and Max Depth = 100) [27]. The genes in clusters with scores > 10 were subsequently performed functional enrichment analysis.

### Weighted gene co-expression network analysis

The “WGCNA” package in R was applied for weighted gene co-expression network analysis [28]. The optimal values of the weighted parameters of the adjacent functions were obtained using the pickSoftThreshold function and were used as soft thresholds for subsequent network construction. Then, a weighted adjacency matrix was constructed, and a hierarchical clustering based on a topological overlap matrix (TOM) with a dissimilarity measure (1-TOM) was used to construct the relevant gene modules. Finally, the correlation of each module with the disease was calculated, and the module with the highest correlation was defined as the key module. The genes within the key modules were screened for subsequent analysis.

### Identification and validation of key crosstalk genes

CytoHubba is a Cytoscape plugin app that can screen hub genes in PPI networks [29]. Genes that generally ranked top 20 in four algorithms (Degree, EPC, MCC, and MNC) were identified as PPI key genes. Subsequently, PPI key genes and key module genes were intersected. The intersections were defined as key crosstalk genes, which were subsequently validated in GSE100927 and GSE10334. We examined the diagnostic effectiveness of the key crosstalk genes with the receiver operating characteristic curves (ROCs) using the “pROC” package in R [30]. In addition, the mRNA expression levels of the key crosstalk genes were compared between case and control groups using an independent t-test, with a P-value of less than 0.05 considered statistically significant. Relative expression levels of the key crosstalk genes in case and control groups were visualized by boxplots through the “ggplot2” R package [31].

### Validation of key crosstalk genes in independent external datasets

To improve the confidence, we validate the expression of the key crosstalk genes in the GSE43292, GSE16134, and GSE28829 datasets. GSE43292 was generated using GPL6244 [HuGene-1_0-st] Affymetrix Human Gene 1.0 ST Array, including 32 carotid atherosclerotic lesion samples and 32 normal carotid samples. GSE16134 was obtained from GPL570 [HG-U133_Plus_2] Affymetrix Human Genome U133 Plus 2.0 Array, which contained 241 gingival tissue samples affected by PD and 69 unaffected control gingival tissue samples. GSE28829 was generated using GPL570 [HG-U133_Plus_2] Affymetrix Human Genome U133 Plus 2.0 Array, including 16 advanced atherosclerotic plaque samples and 13 early atherosclerotic plaque samples from carotid arteries.

### Immune infiltration analysis

The expression levels of immune cells for each sample of GSE10334 and GSE100927 were quantified using the ssGSEA algorithm from the GSVA R package [32]. The gene set of the cell marker for immune cells was derived from a previous study by Charoentong and colleagues [33]. We then calculated the correlation between the expression of key crosstalk genes and the expression of immune cells in the samples of case groups in GSE10334 and GSE100927 using the Spearman method.

### Identification of transcription factors (TFs) of key crosstalk genes

First, we predicted TFs of the key crosstalk genes through the NetworkAnalyst 3.0 platform (https://www.networkanalyst.ca/) [34]. Then, TFs interacting with at least two key crosstalk genes were screened. Subsequently, we validated the mRNA expression levels of these TFs in the test set (GSE100927 and GSE10334) and the validation set (GSE43292, GSE16134, and GSE28829) by independent t-tests. Finally, TFs that were commonly upregulated in case groups were identified as potential key TFs in CAS and PD.

## Results

### Identification of overlapping DEGs of CAS and PD

**Fig. 1:**
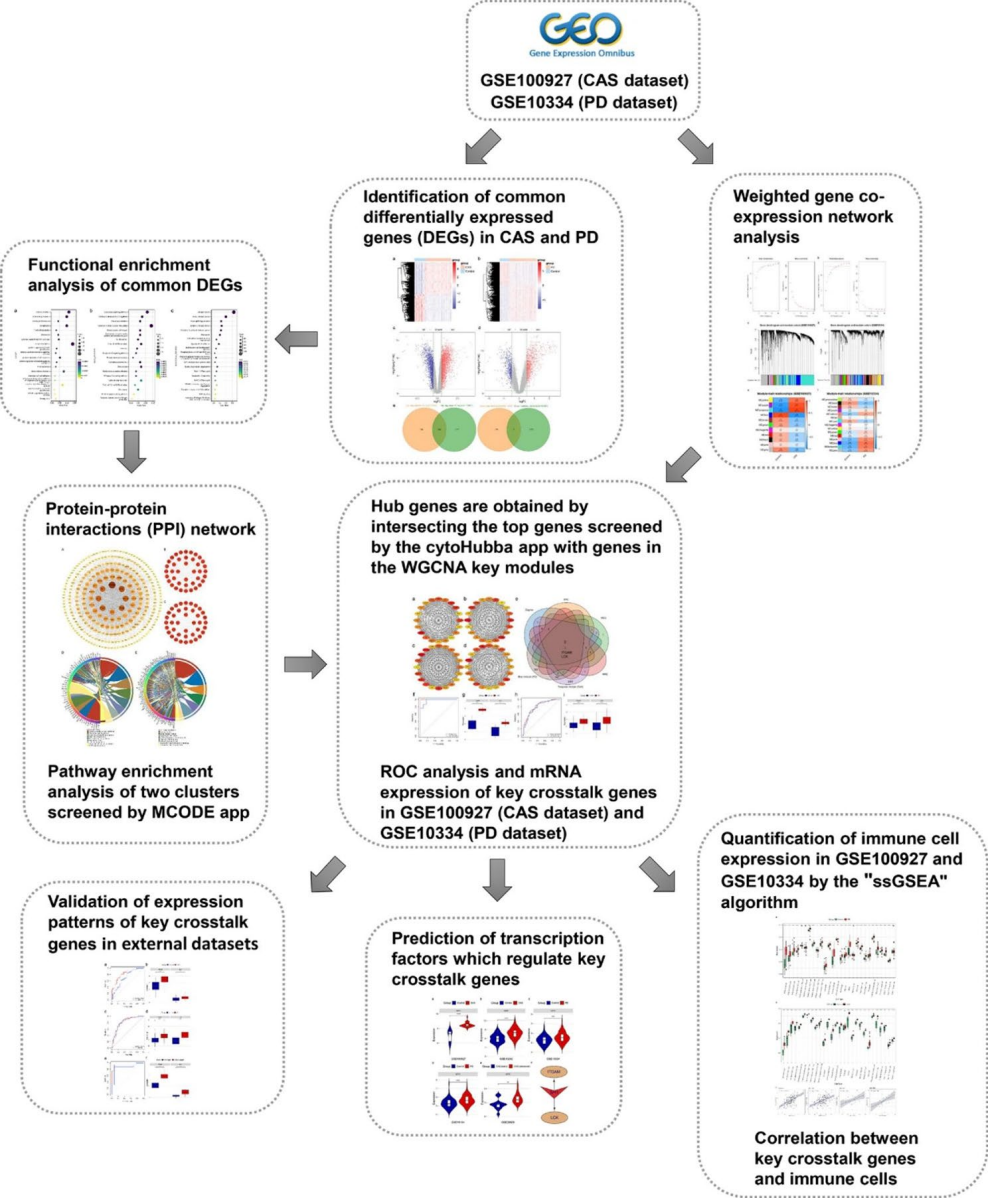
The flow diagram for the whole study

**Fig. 2:**
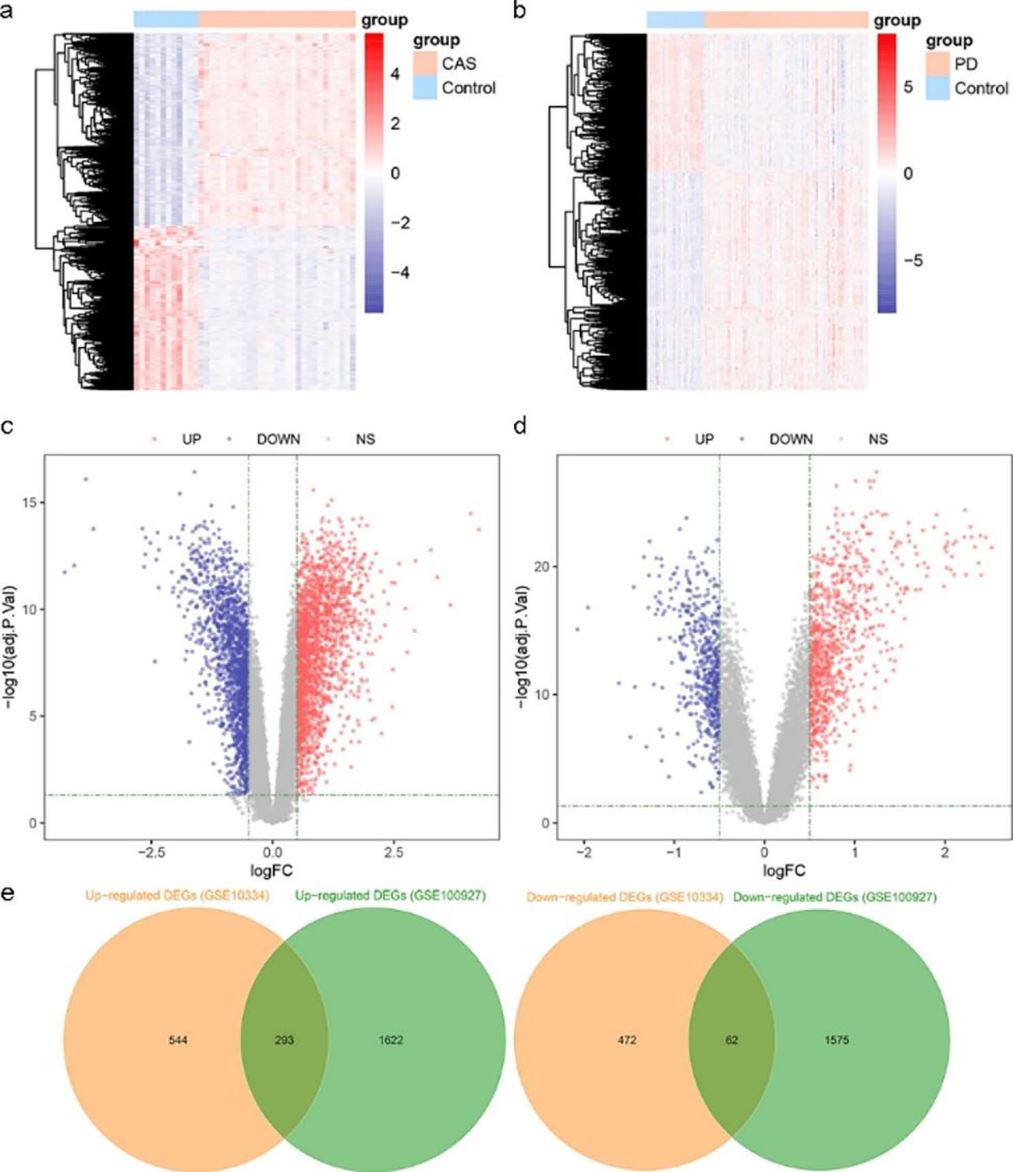
DEGs in GSE100927 and GSE10334. (a) Heatmap of GSE100927. (b) Heatmap of GSE10334. (c) Volcano map of GSE100927. (d) Volcano map of GSE10334. (e) Veen diagrams indicate that GSE100927 and GSE10334 share 293 overlapping up-regulated DEGs and 62 overlapping down-regulated DEGs.

Figure 1 shows the flow diagram for this study. There were 3552 DEGs identified in GSE100927, of which 1915 were up-regulated, and 1637 were down-regulated (Fig. 2a and c). In addition, 1371 DEGs were identified in GSE100927, including 837 up-regulated genes and 534 down-regulated genes (Fig. 2b and d). Through Veen diagrams, we identified 293 overlapping up-regulated DEGs and 62 overlapping down-regulated DEGs between GSE100927 and GSE10334 (Fig. 2e).

### Functional enrichment analysis of overlapping DEGs

We conducted functional enrichment analyses of overlapping DEGs in the DAVID database. The GO_BP analysis revealed that the most significantly enriched terms were immune response, inflammatory response, neutrophil chemotaxis, cell adhesion, and T cell differentiation. KEGG analysis showed that the overlapping genes were likely related to chemokine signaling pathway, leukocyte transendothelial migration, rheumatoid arthritis, cytokine-cytokine receptor interaction, and hematopoietic cell lineage. Reactome analysis indicated that overlapping DEGs were significantly enriched in immune system, innate immune system, neutrophil degranulation, adaptive immune system, and cytokine signaling in immune system. Figure S1 illustrates the top 20 terms that were significantly enriched in GO_BP, KEGG pathway, and Reactome pathway.

### Construction of PPI networks with overlapping DEGs

**Fig. 3:**
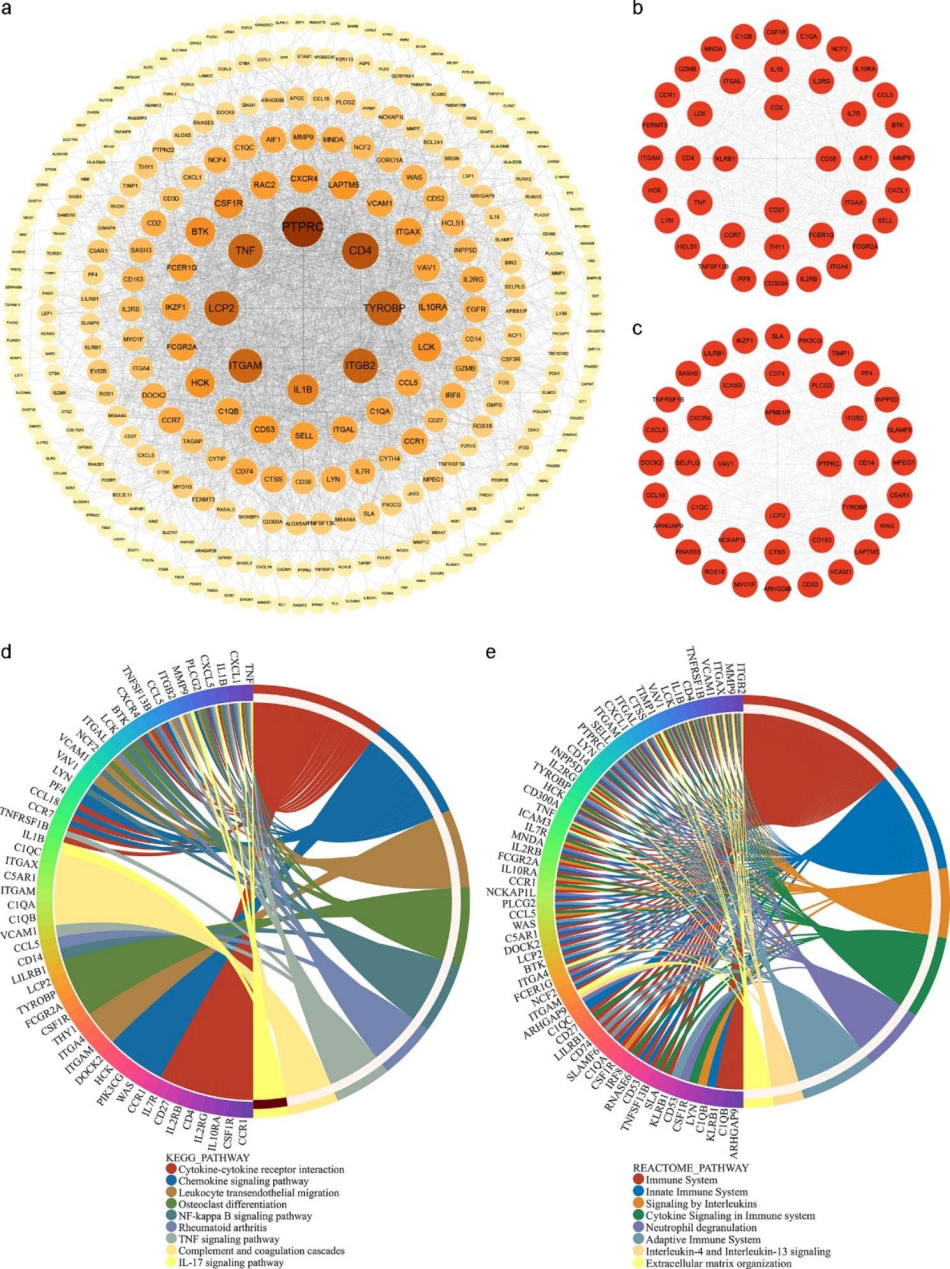
PPI network of overlapping DEGs. (a) PPI network includes 302 nodes and 2844 interaction pairs. (b)-(c) Two gene clusters with high connectivity obtained by the MCODE algorithm in the PPI network. (d) KEGG pathway enrichment analysis of genes in two gene modules. (e) Reactome pathway enrichment analysis of genes in two gene modules

A PPI network with 302 nodes and 2844 interaction pairs was constructed from the STRING database. The PPI network was visualized using the Cytoscape software (Fig. 3a). The MCODE plugin app identified two clusters with high connectivity (scores > 10) (Fig. 3b and c). KEGG analysis showed that two clusters were significantly enriched in cytokine-cytokine receptor interaction, chemokine signaling pathway, leukocyte transendothelial migration, osteoclast differentiation, NF-kappa B signaling pathway, rheumatoid arthritis, TNF signaling pathway, complement and coagulation cascades, and IL-17 signaling pathway (Fig. 3d). Reactome analysis revealed that two clusters were mainly involved in immune system, innate immune system, signaling by interleukins, cytokine signaling in immune system, neutrophil degranulation, adaptive immune system, interleukin-4 and interleukin-13 signaling, and extracellular matrix organization (Fig. 3e).

### Construction of weighted gene co-expression network and screening of key modules

**Fig. 4:**
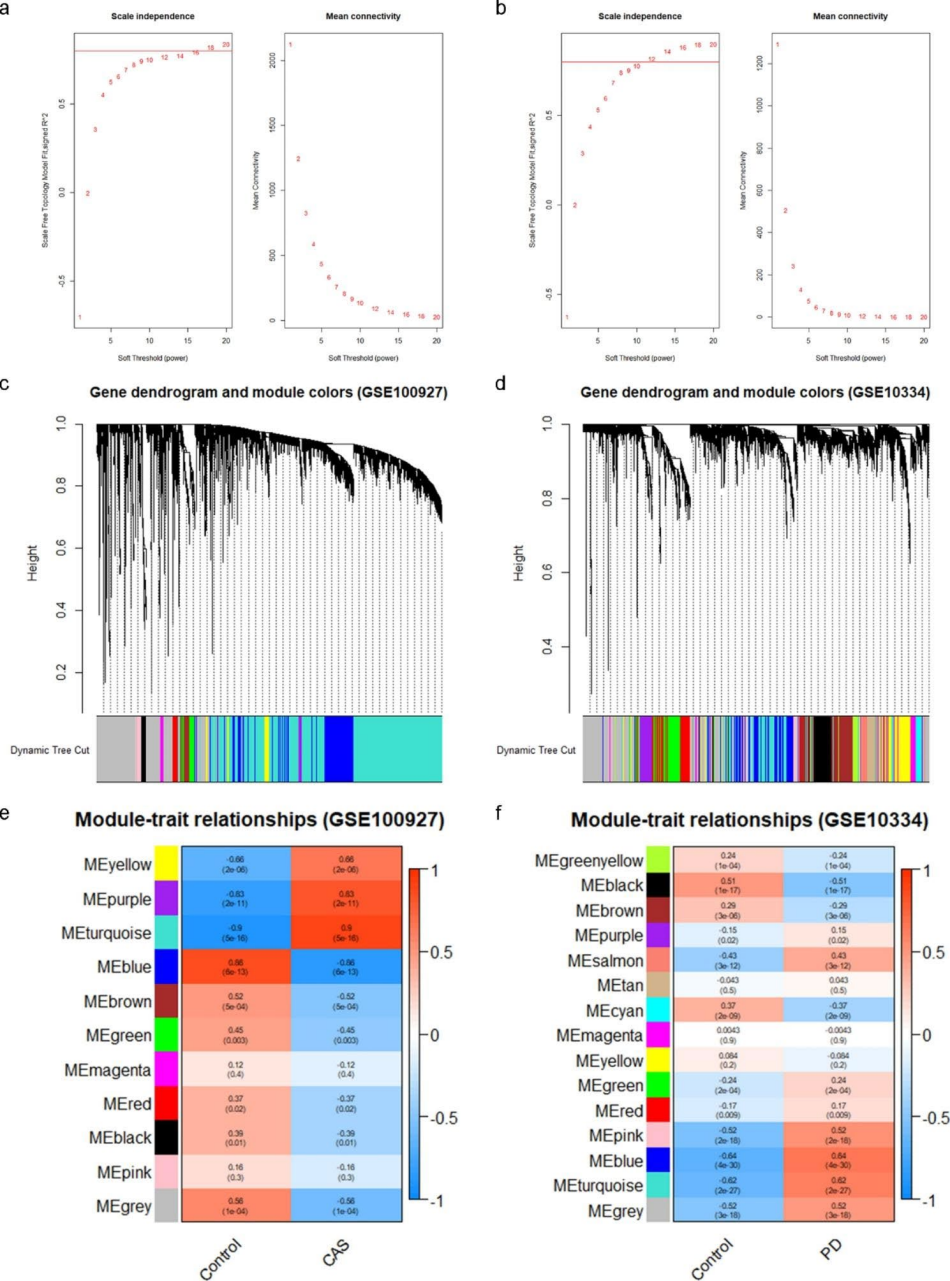
Weighted gene co-expression network analysis (WGCNA). (a) Soft threshold determination in GSE100927 (CAS dataset). (b) Soft threshold determination in GSE10334 (PD dataset). (c) Hierarchical clustering dendrograms of the top 5000 genes with the highest standard deviation in the CAS clustered based on a dissimilarity measure (1-TOM). (d) Hierarchical clustering dendrograms of the top 5000 genes with the highest standard deviation in the PD clustered based on a dissimilarity measure (1‐TOM). (e) Module–trait relationships in CAS. Each different colored module contains the corresponding correlation and p-value. (f) Module–trait relationships in PD. Each different colored module contains the corresponding correlation and p-value

The top 5000 genes with the highest standard deviation of expression were screened for WGCNA. The value of β = 18 and β = 12 (scale-free R2 = 0.80) were selected as the soft-threshold power of CAS and PD respectively to ensure scale-free networks (Fig. 4a and b). With minModuleSize set to 40, 11 modules were identified in GSE100927, and 15 modules were identified in GSE10334 (Fig. 4c and d). Different colors indicated different modules. Finally, based on Pearson correlation coefficients, heat maps on module-trait relationships were generated to assess the association between each module and the disease. As a result, the turquoise module was most correlated with CAS (0.9, p = 5E-16), containing 2441 genes, and the blue module was most correlated with PD (0.64, p = 4E-30), including 555 genes (Fig. 4e and f). The genes in these two modules were subsequently analyzed to screen for key crosstalk genes.

### Identification and validation of key crosstalk genes

**Fig. 5:**
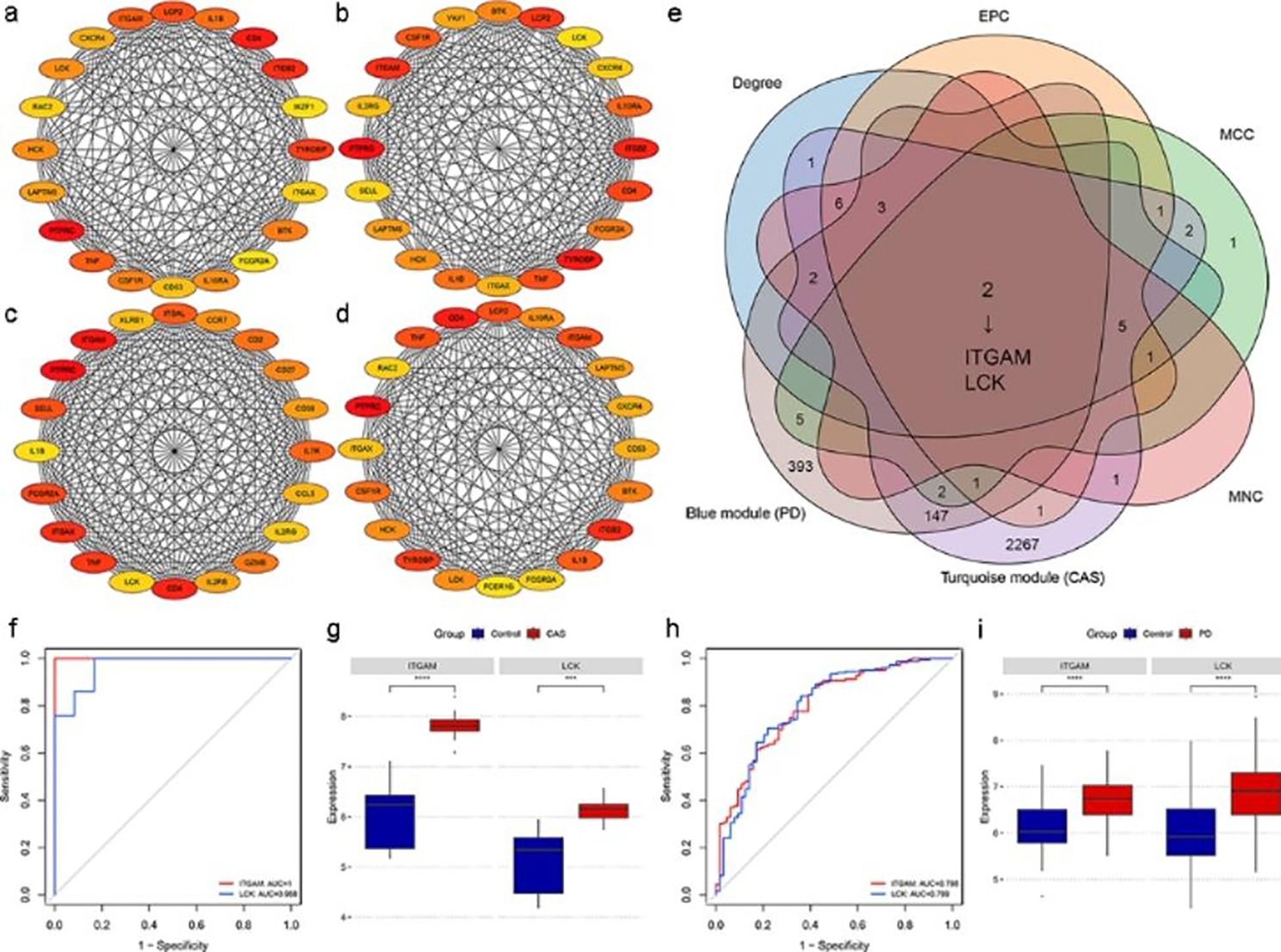
Identification and validation of key crosstalk genes. (a)-(d) The top 20 genes in PPI networks ranked by Degree, EPC, MCC, and MNC, respectively. (e) ITGAM and LCK existed both in the top 20 genes of the four algorithms and in the two key modules. (f) ROC curve analysis of the key crosstalk genes in GSE100927. (g) Expression levels of the key crosstalk genes in GSE100927. (h) ROC curve analysis of the key crosstalk genes in GSE10334. (i) Expression levels of the key crosstalk genes in GSE10334. ^***^ p < 0.001; ^****^ p < 0.0001

CytoHubba plug-in of Cytoscape was used to calculate the top 20 genes based on four algorithms (Degree, EPC, MCC, and MNC) (Fig. 5a, b, c, and d). Table S1 presents the top 20 genes calculated by the four algorithms. The Veen diagram showed that ITGAM and LCK existed both in the top 20 genes of the four algorithms and in the two key modules (Fig. 5e). Therefore, these two genes were identified as key crosstalk genes. According to the ROC curves, LCK and ITGAM were effective for diagnosing CAS and PD (Fig. 5f h). In addition, the mRNA expression levels of the key crosstalk genes were significantly upregulated in the case groups of GSE100927 and GSE10334 (Fig. 5g, 5i).

### Validation of the key crosstalk genes in independent external datasets

**Fig. 6:**
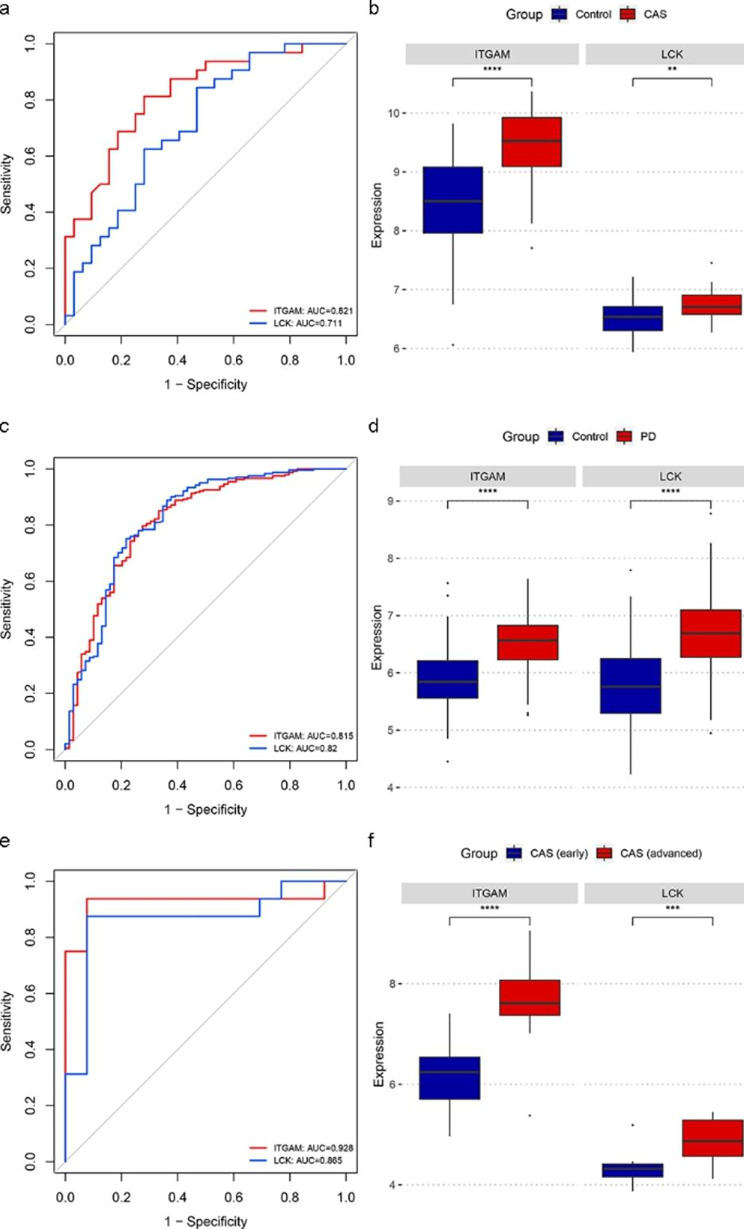
Validation of the key crosstalk genes in independent external datasets. (a) ROC curve analysis of key crosstalk genes in GSE43292. (b) Expression levels of key crosstalk genes in GSE43292. (c) ROC curve analysis of key crosstalk genes in GSE16134. (d) Expression levels of key crosstalk genes in GSE16134. (e) ROC curve analysis of key crosstalk genes in GSE28829. (f) Expression levels of key crosstalk genes in GSE28829. ^**^ p < 0.01; ^***^ p < 0.001; ^****^ p < 0.0001

To further verify the efficacy of the key crosstalk genes. We validated the genes in two additional independent external datasets (GSE43292 and GSE16134). Consistent with the results of the test set, the ROC results showed good diagnostic efficacy in the external dataset (Fig. 6a and c), and the mRNA expression levels of the key crosstalk genes elevated in case groups of two external datasets (Fig. 6b and d). In addition, in the GSE28829 dataset, ROC results suggested the effectiveness of LCK and ITGAM in discriminating between advanced and early atherosclerotic plaques (Fig. 6e), and the expression of the key crosstalk genes was significantly higher in advanced atherosclerotic plaque samples in comparison to early plaque samples (Fig. 6f), indicating that LCK and ITGAM are strongly associated with CAS severity.

### Immune infiltration analysis

**Fig. 7:**
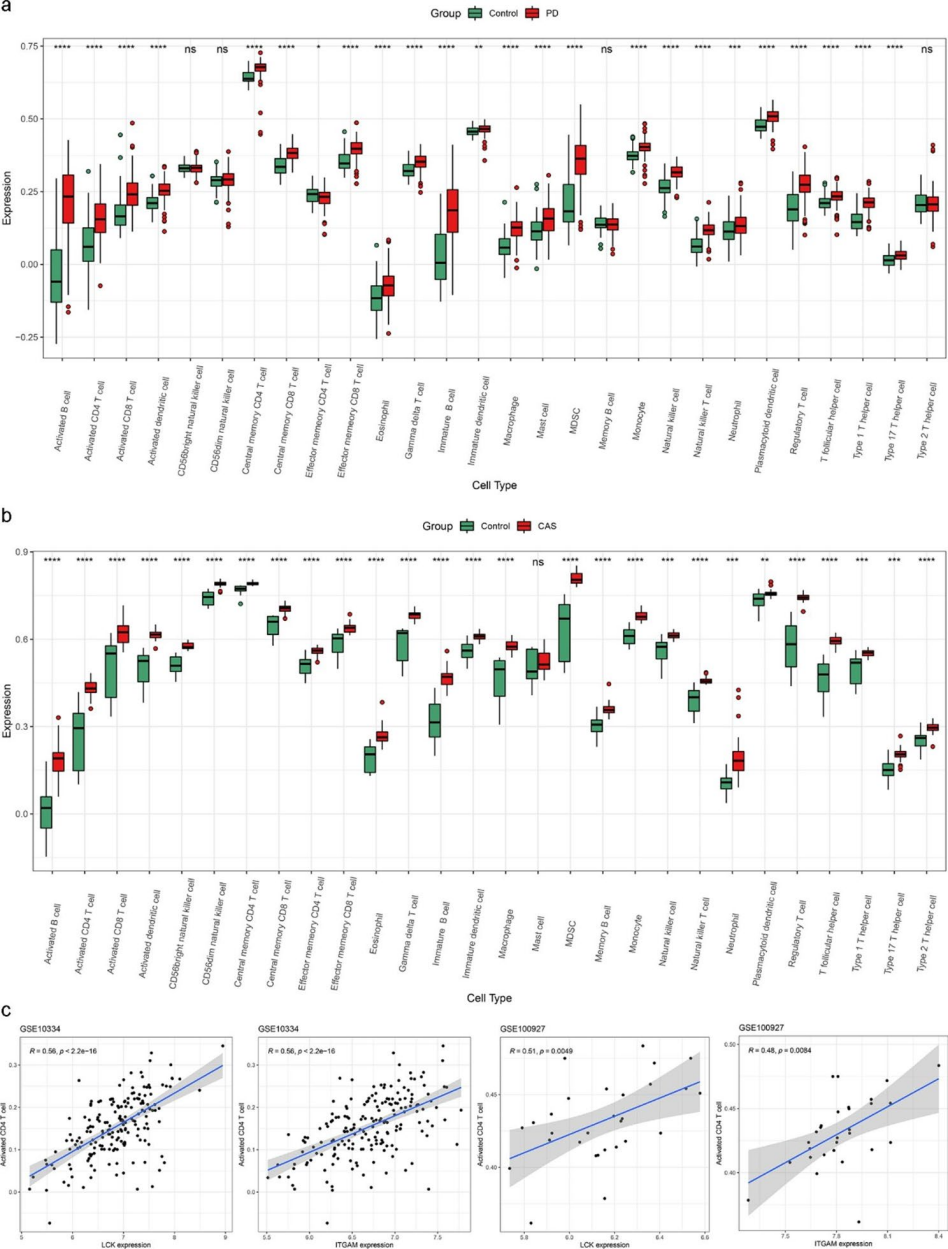
Results of immune infiltration analysis. (a) Boxplots of the expression of each immune cell between PD and control in the GSE10334 dataset. (b) Boxplots of the expression of each immune cell between CAS and control in the GSE100927 dataset. (c) The expression of two key crosstalk genes was significantly correlated with the expression of activated CD4 T cells in both GSE10334 and GSE100927 datasets. ^*^ p < 0.05; ^**^ p < 0.01; ^***^ p < 0.001; ^****^ p < 0.0001

Through enrichment analysis, we found that the immune pathway is involved in crosstalk between PD and CAS; therefore, we calculated the content of each immune cell in the case group versus the control group in both datasets using the ssGSEA algorithm. Figure 7a and b indicated that the immune landscape is considerably altered in PD and CAS compared to the control group. Furthermore, correlation analysis showed that two key crosstalk genes (LCK and ITGAM) were both significantly associated with the expression of activated CD 4 T cells (Fig. 7c).

**Fig. 8:**
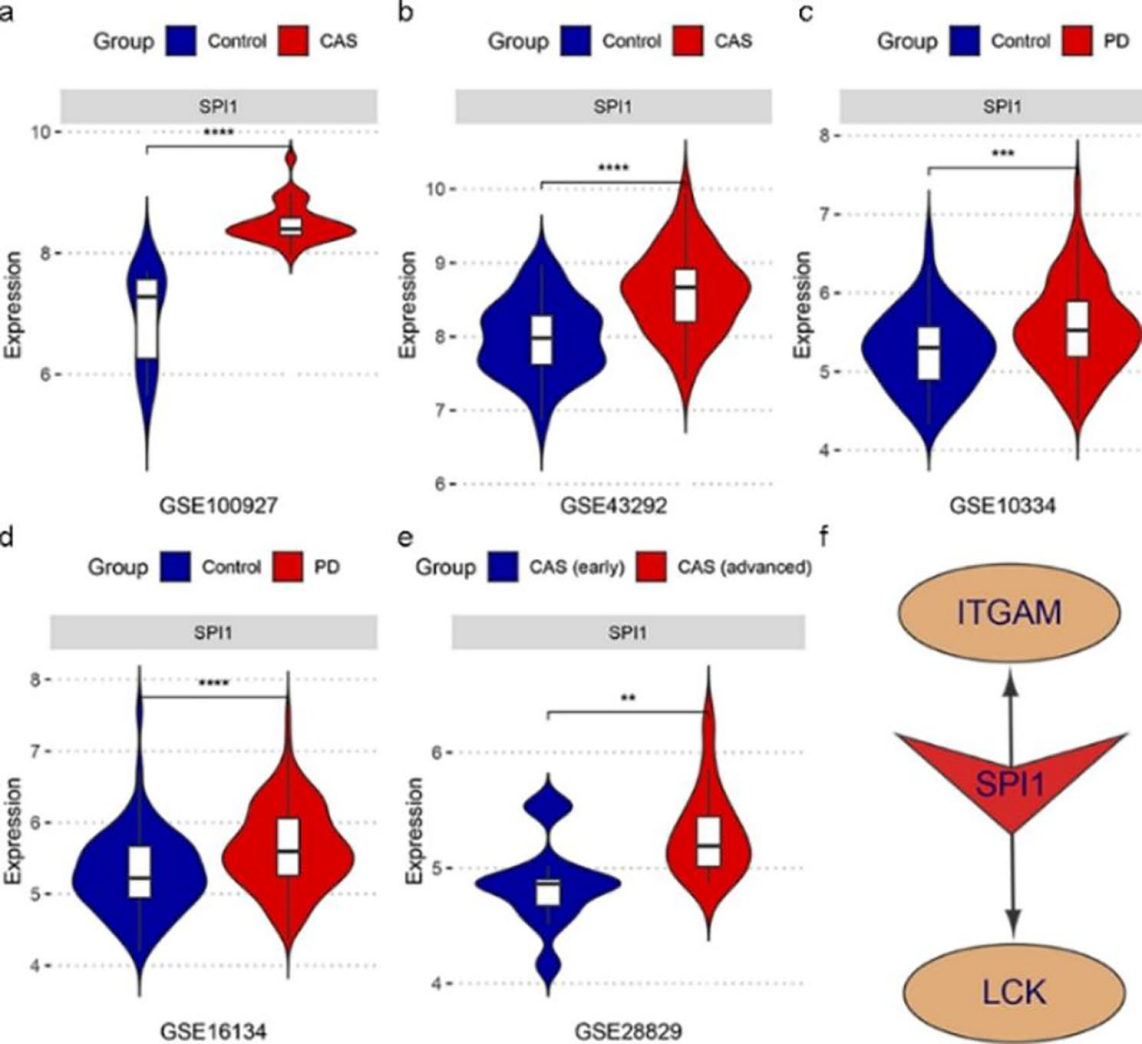
SPI1 was identified as a shared potential key TFs in CAS and PD. (a)-(d) The mRNA expression level of SPI1 was generally up-regulated in the case groups in GSE100927, GSE43292, GSE10334, and GSE16134. (e) The mRNA expression level of SPI1 was significantly higher in advanced atherosclerotic plaque samples compared to early plaque samples. (f) The potential TF regulatory network in CAS and PD. ^**^ p < 0.01; ^***^ p < 0.001; ^****^ p < 0.0001

### Exploring key transcription factors (TFs) regulating the key crosstalk genes

We explored the potential TFs that may regulate the key crosstalk genes. According to the results generated by NetworkAnalyst 3.0, we screened TFs that can regulate two key crosstalk genes (ITGAM and LCK) and calculated their mRNA expression levels in all datasets included in our study by independent t-tests. Consequently, the expression levels of SPI1 elevated in all case groups in GSE100927, GSE43292, GSE10334, and GSE16134 (Fig. 8a and d). In addition, in the GSE28829 dataset, the expression level of SPI1 was significantly higher in advanced atherosclerotic plaque samples in comparison to early plaque samples (Fig. 8e). Therefore, SPI1 might be a potential key TF regulating the two key crosstalk genes (ITGAM and LCK) in the pathological process in CAS and PD (Fig. 8f).

**Fig. 9:**
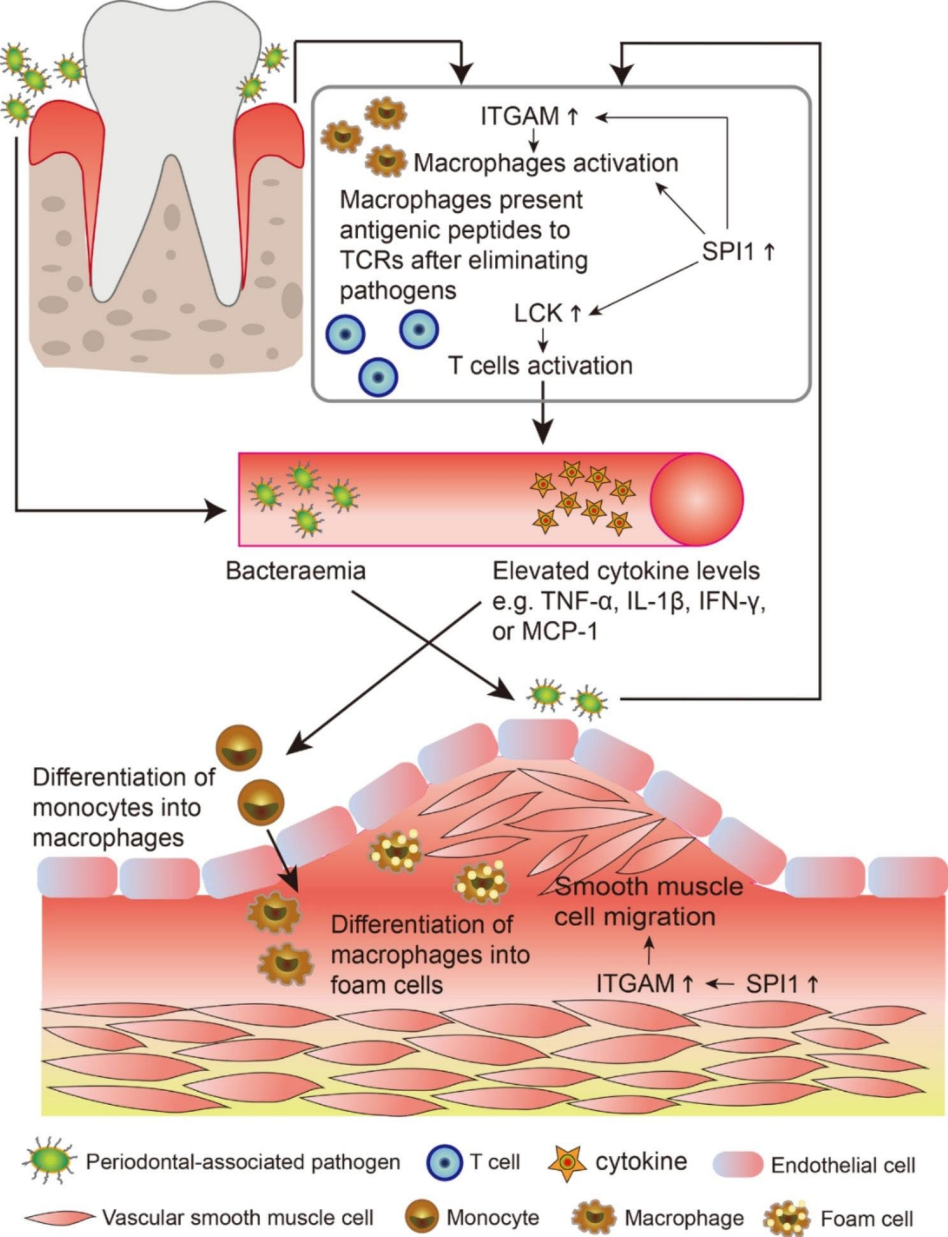
PD and CAS co-morbidity mechanisms involving the key crosstalk genes and TF.

## Discussion

In a study by Ning and colleagues, gene expression profiles of peripheral blood mononuclear cells (PBMCs) obtained from patients with PD or CAS were analyzed [35]. Another study by Trindade et al., incorporating bibliometric analysis and the DisGeNET database, identified the dysregulated molecules, including C-reactive protein, interleukin-6 and 1-beta, myeloperoxidase, and matrix metalloproteinase-9, as important mediators of periodontitis and coronary artery disease [36]. The DisGeNET database, which was utilized in these two studies, allows users to obtain genes relevant to particular diseases [37]. However, this approach may filter out some potentially valuable molecules. Therefore, we performed analysis directly using transcriptomic data of PD and CAS and finally identified key crosstalk genes and TFs, as well as their mechanisms leading to PD and CAS co-morbidity from an immune and inflammatory perspective.

By comprehensive analysis of gene expression profiles, LCK and ITGAM were identified as two key crosstalk genes between CAS and PD. LCK is a member of the Src family of protein tyrosine kinases (PTKs). He et al. identified an essential role for LCK in the imbalance of the immune system in periodontitis through bioinformatics analysis [38]. LCK inhibitor attenuates the development of atherosclerosis and promotes plaque stability [39]. In addition, LCK was reported to inhibit heat shock protein 65-mediated reverse cholesterol transport in T cells, which is involved in one of the causes of atherosclerosis [40]. ITGAM encodes the integrin alpha M chain. Integrins expressed in periodontal tissues are involved in regulating cell attachment, maintaining tissue integrity, and mediating cell signaling, gene expression, and cytokine activation [41]. In addition, it was shown that ITGAM was significantly increased in the gingival tissue of the aged nonhuman primate model [42]. A proteomics study has revealed a significant elevation of ITGAM in gingival tissue of chronic periodontitis patients [43]. Integrin signaling has been associated with multiple aspects of atherosclerosis, including the early induction of inflammation and the development of advanced fibrotic plaques [44]. Zhou et al. identified that high ITGAM level was associated with atherosclerotic plaque instability and poor outcomes in ischemic stroke [45]. Mass spectrometry analysis identified integrin Alpha-M in emboli from patients with high LDL [46]. In addition, ITGAM has been identified as one of the crosstalk genes for atherosclerosis and COVID-19 co-morbidity [47]. Therefore, the two upregulated key crosstalk genes (ITGAM and LCK) might be involved in CAS and PD co-morbidity mechanisms.

Enrichment analysis of crosstalk genes indicated that immune pathways and inflammatory pathways participated in the co-morbidity of PD and CAS. In addition, immune cell infiltration analysis revealed significantly different immune patterns in the disease groups of PD and CAS compared to controls. Two key crosstalk genes were highly correlated with activated CD4 T cells. Thus these two key crosstalk genes may contribute to PD and CAS co-morbidities through immune and inflammatory pathways.

The gum tissue of individuals who neglect oral hygiene may be vulnerable to pathogens such as Porphyromonas gingivalis [48]. Macrophages eliminate pathogenic microorganisms and present antigenic peptides to the T cell receptor (TCR) [49]. The critical role of LCK in TCR signaling and its involvement in T cell activation was previously reported [50]. Thus, the upregulated LCK in PD is partially involved in T cell activation, which initiates the adaptive immune response. Subsequently, levels of circulating cytokines (such as IL-1β, TNF-α, IFN-γ, and MCP-1) rise, elevating the global inflammation level and promoting the conversion of macrophages into foam cells at the artery, ultimately leading to plaque formation [51, 52]. Furthermore, pathogens that reach the damaged endothelium of the carotid artery as a result of bacteremia brought about by periodontitis can increase LCK in the carotid vessels, which can produce the inflammatory and immune effects mentioned above and exacerbate the CAS process.

Integrin ITGAM/ITGB2 is implicated in various adhesive interactions of monocytes, macrophages, and granulocytes, as well as in mediating the uptake of complement-coated particles and pathogens [53]. Studies have shown that integrin activation can promote smooth muscle cell proliferation and macrophage infiltration, consequently aggravating atherosclerosis progression [54]. A recent study by Zhou et al. identified that ITGAM might promote the growth and progression of abdominal aortic aneurysms by promoting endothelial cell adhesion and the migration of circulating monocytes and macrophages [55]. Therefore, ITGAM activation resulting from alterations in the gingival microenvironment following a pathogenic attack may be partially involved in the local macrophage activation, consequently enhancing the inflammatory response. In addition, the altered local vascular environment of CAS induces ITGAM upregulation, resulting in the differentiation of monocytes to macrophages, promoting macrophage activation and smooth muscle cell migration to the endothelium, further exacerbating the progression of CAS.

Our study revealed that SPI1, encoding the transcription factor PU. 1, is commonly upregulated in PD and CAS, which regulates key crosstalk genes. Despite few studies reporting specific functions of PU.1 in PD and CAS, there is evidence that PU. 1 is a key regulator of cellular communication in the immune system, capable of modulating cytokines and cytokine receptors regulating inflammation [56]. In addition, PU.1 specifically participates in macrophage activation [57]. Thus in the global inflammatory environment of PD and CAS, SPI1 (PU. 1) may simultaneously activate macrophages and regulate LCK and ITGAM levels. Figure 9 demonstrates the potential role of the key crosstalk genes and TF identified by our study in the mechanism of PD and CAS co-morbidity.

Our study identifies key crosstalk genes and TF in PD and CAS from an immune and inflammatory perspective, thus providing new ideas on the co-morbidity mechanisms of PD and CAS. The significance of this study is to remind people especially those with high cardiovascular disease risk factors and those who suffer from CAS to focus on oral hygiene. It should be noted that the conclusions were obtained by combining bioinformatics analysis and the previous relevant study findings, thus more clinical validation is required in the future. In addition, the specific function of the crosstalk genes remains for validation in vivo and in vitro, for instance whether perturbation of the crosstalk genes on APOE^−/−^ periodontitis mice/rats will alter CAS progression.

## Conclusion

In summary, we identified shared DEGs of CAS and PD, performed enrichment analysis, constructed PPI networks, and conducted WGCNA. Eventually, ITGAM and LCK were identified as key crosstalk genes for CAS and PD, and they may be involved in the crosstalk between CAS and PD through immune pathways and inflammatory pathways. In addition, SPI1 was identified as a potential key TF in CAS and PD. This study provides new insights into the co-pathogenesis of CAS and PD, and the present findings need further validation in the future.

## Electronic supplementary material

Below is the link to the electronic supplementary material.

Supplementary Material 1

Supplementary Material 2

## Data Availability

The datasets analyzed in this study are available for download in the Gene Expression Omnibus (GEO) database, https://www.ncbi.nlm.nih.gov/geo/, (GSE100927, GSE10334, GSE43292, GSE16134, and GSE28829).

## Abbreviations

CAS: Carotid Atherosclerosis.
PD: Periodontitis.
GEO: Gene Expression Omnibus.
DEG: Differentially expressed gene.
PPI: Protein-protein Interaction.
WGCNA: Weighted Gene Co-expression Network Analysis.
TF: Transcription factor.
