# Supplementary material for: Integrated analysis and exploration of potential shared gene signatures between carotid atherosclerosis and periodontitis
Source: BMC Med Genomics. 2022 Oct 31;15:227. doi: 10.1186/s12920-022-01373-y (PMC9620656; doi:10.1186/s12920-022-01373-y)
Supplement: Supplementary file 2 — Supplementary Material 2 [file 12920_2022_1373_MOESM2_ESM.pdf]

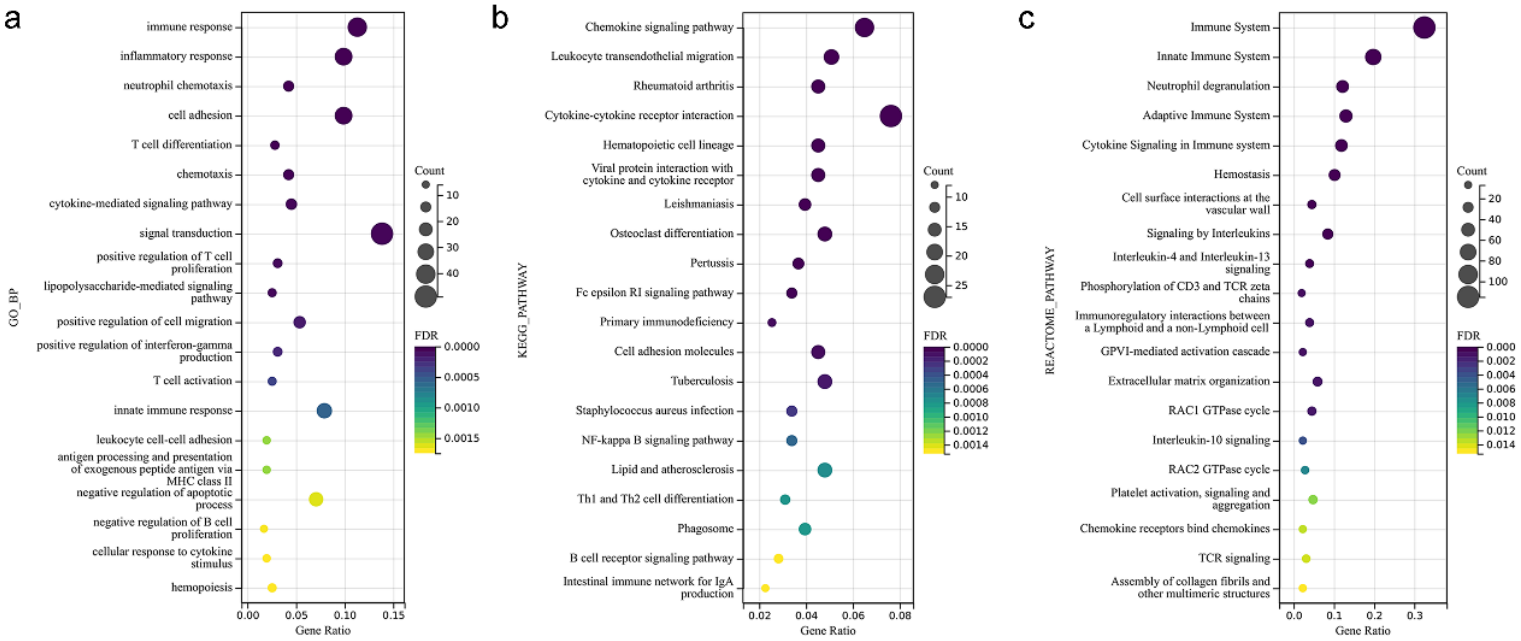

Figure S1 Functional enrichment analysis of overlapping DEGs. (a) The top 20 significant enriched terms for Gene Ontology biological process (GO_BP). (b) The top 20 significant enriched terms for Kyoto Encyclopedia of Genes and Genomes (KEGG) pathway. (c) The top 20 significant enriched terms for Reactome pathway.
